# Prevalence and predictors of postpartum depression among women attending clinics in Gaborone

**DOI:** 10.4102/sajpsychiatry.v31i0.2373

**Published:** 2025-07-18

**Authors:** Angelina M. Mannathoko, Keneilwe Molebatsi, Deogratias O. Mbuka

**Affiliations:** 1 Department of Family Medicine and Public Health, Faculty of Medicine, University of Botswana, Gaborone, Botswana; 2 Department of Psychiatry, Faculty of Medicine, University of Botswana, Gaborone, Botswana; 3 Department of Public Health and Family Medicine, Faculty of Medicine, University of Botswana, Gaborone, Botswana

**Keywords:** postpartum depression, maternal mental health, Botswana, risk factors, prevalence, protective factors

## Abstract

**Background:**

Untreated postpartum depression (PPD) has the potential to cause significant distress or impairment in functioning with a consequent negative impact on a developing child.

**Aim:**

This study aimed to determine the prevalence of PPD and its associated factors in women attending postpartum primary care clinics.

**Setting:**

The study setting involved randomly selected three 24-h clinics in Gaborone, the capital city of Botswana.

**Methods:**

A cross-sectional study was conducted among 295 conveniently sampled postpartum mothers. The Edinburgh Postnatal Depression Scale (EPDS) questionnaire, researcher-designed socio-demographic questions and the Oslo Social Support Scale 3 (OSSS-3) were utilised to collect data on the PPD, demographic factors and social support, respectively. Variables identified to be associated with PPD on bivariate analyses were entered into multivariate analysis to determine factors associated with PPD.

**Results:**

The prevalence of PPD was 33.9% (95% CI 28.5% – 39.6%). Factors predictive of PPD included the history of being involved in intimate partner violence (AOR = 4.789 95% CI [2.276–10.077]), poor relationship with the partner’s mother (AOR 2.657, [1.080–6.538]), poor and moderate social support (AOR 2.685 [1.013–7.111] and AOR 5.897 [2.140–16.248]), respectively.

**Conclusion:**

The high prevalence of PPD highlights the need for routine screening for PPD and its associated factors in antenatal and postnatal clinics. Continued practice of traditional postpartum cultural practices can be recommended as these promote social support and can potentially decrease PPD in our setting.

**Contribution:**

This is the first study to report on the prevalence and factors associated with PPD in Botswana, thus useful in tailoring culturally appropriate interventions.

## Introduction

Postpartum depression (PPD) is defined as an episode of depression with onset of symptoms 4 weeks and up to 1 year following delivery with no psychotic features.^1,2,3^ The signs and symptoms are generally the same as those of major depression occurring at any different times in life.^2^ Postpartum depression is one of the common complications in the postpartum period, and if untreated, it has the potential to result in profound negative outcomes affecting the mothers, children and their families,^4^ making it a significant health problem that requires attention.

A systemic review 291 PPD prevalence studies from 56 countries found that the global pool prevalence of PPD was approximately 17.7%, with significant variations. Chile is a less developed country, having the highest prevalence of 38% PPD and Singapore is a well-developed country, having one of the lowest prevalence of 3% PPD.^5^ There has been a steady rise in the global prevalence of PPD when comparing the above pooled prevalence to the 13% estimated in a meta-analysis by O’Hara MW and Swain^6^ in 1996, indicating that there is a rise in mental health challenges among postnatal mothers.

In sub-Saharan Africa, PPD among adult women ranged from 33.2% in Southwest Ethiopia,^7^ which is higher compared to the western world, to as low as 9.2% in Sudan^8^ and an even lower prevalence of 7% in Ghana.^9^ In Southern Africa, a study conducted in urban Johannesburg, South Africa, found that 24% of mothers at 6 months post-delivery had PPD,^10^ while Stellenberg and colleagues^11^ reported that in rural South Africa, the prevalence of PPD ranged from 48% to 50.3%, with 28% of their participants having severe PPD. In Eswatini, 47.4% of the participants screened positive for PPD, of whom 19% had thoughts of harming themselves and/or infanticidal ideation during the postpartum period.^12^ Findings from these studies demonstrate that the prevalence of PPD is high in African countries such as Botswana.

The actual cause of PPD is not known but hormonal factors, genetics and immune function have evidently been found to play a role in the development of PPD.^4^ There are expeditious changes in levels of oestrogen and progesterone in the postpartum period, and these play an important role in emotion processing, arousal, cognition and motivation of mothers in the postpartum period. A genetic variation on chromosome 1q21.3–q32.1 and 9p24.3–p22.3 and in Hemicentin-1 (HMCN1), which contains several oestrogen-binding sites, was found in 1200 women, and it appeared to increase the susceptibility of developing PPD.^4^

Globally, factors that have been found to predispose new mothers to developing PPD include a history of depression or anxiety before pregnancy and antepartum, previous premenstrual dysphoria, stressful life events during pregnancy, poor social support, marital conflict, low income, immigrant status, a lack of postnatal parental competence, low parenting self-efficacy and young maternal age.^13,14^

Most of the risk factors for PPD in developing countries are similar to those identified in developed nations.^13^ A review of factors contributing to the development of PPD in Africa revealed that the odds of having PPD were higher among women with poor obstetric history such as hyperemesis gravida, caesarean section delivery, adverse birth and infant health outcomes. Exposure to different forms of intimate partner violence (IPV) was also associated with the occurrence of PPD.^15^ Poor maternal health conditions, for example, hypertension, gestational diabetes, or human immunodeficiency virus and/or acquired immunodeficiency syndrome and tuberculosis (HIV and/or AIDS, TB) were also found to be significant risk factors for PPD.^14^

Women with inadequate social support, a short inter-pregnancy interval, high levels of family stress and a perceived economic hardship have also been found to be at increased risk of PPD.^16^ In Ethiopia, conflict with partner, poor relationship with partner’s mother, partner not helping with childcare, unplanned pregnancy, age greater than 30 years or a chronic physical illness, death of infant and current marital problems were reported to have played a decisive role in the development of PPD.^7^ Closer to Botswana, evidence from Zimbabwe and South Africa suggests similar risk factors, such as food insecurity, intimate partner violence, poor obstetric outcomes, being unmarried, unplanned pregnancy, unemployment, having a history of a psychiatric illness, poor support and problematic drinking during pregnancy.^11,17,18^ Knowledge of these risk factors is of considerable importance as it can aid early detection and identification of women at risk of developing PPD in our setting.

A systematic review by Slomian and colleagues^19^ states that PPD is associated with negative effects on maternal physical and psychological health, quality of life, and ability to bond and interact with their infant, partner and relatives. They also indicated that maternal PPD is associated with high-risk behaviours such as smoking and an increased prevalence of suicidal ideations, alcohol and illicit drug abuse:

Maternal depression has the ability to compromise the ability of expectant mothers to adopt a healthy lifestyle, impair breastfeeding and disrupt the caregiving roles of mothers during the postpartum period.^9^

This can have a significant impact on the infant and young child’s growth, health and nutritional status. Postpartum depression has also been shown to negatively affect the infant’s cognitive and language development.^20^ In terms of the child’s behaviour, children of affected mothers are more distractible, are antisocial and have neurotic behaviour in the home and at school.^9^ ‘The impact is likely to be worse where depression is severe and prolonged, or when it occurs in the context of adversity’.^9^

The World Health Organization (WHO) considers maternal mental health problems a major public health challenge globally. There is therefore a need to incorporate mental health assessment when delivering services for maternal and child healthcare. This highlights the need to determine the prevalence of PPD, a disorder that impacts not only the mother but also the growing infant and close family members, and its associated factors. Therefore, the aim of this study was to determine the prevalence, protective factors and risk factors of PPD among women attending child wellness clinics in greater Gaborone District Health Management Team (DHMT) clinics.

## Methods

### Study design and setting

A cross-sectional study was conducted in Gaborone, the capital and largest city of Botswana with a population of 244, 107, which is about 10% of the total population of Botswana.^21^ The study was conducted in randomly selected three of the eight 24-h greater Gaborone clinics. A list of clinics that operate for 24 h was compiled and entered in Excel 2019, and a simple random function was used to generate a random list of clinics to be included in the study. The healthcare services provided in these clinics include antenatal and postnatal services for expectant mothers, general outpatient consultations, treatment of minor injuries and under-five childcare services to communities within their reach.

### Study population and sampling strategy

The study population consisted of postnatal mothers coming for their sixth-week check-up and those bringing their children to under-five wellness and immunisation check-ups at the second and third months of the postpartum period. To be included in the study, participants had to sign an informed consent form and be at least 18 years of age. Convenience sampling was used to recruit participants.

The sample size was calculated using the Cochrane formula^22^ considering a 50% prevalence of PPD in urban areas of similar settings as Gaborone and with a 1.96 margin of error and 95% level of significance. The calculated sample size was adjusted to the population size of the three clinics of 662^23^ and rounded up to 280 in anticipation of 15% non-response. Proportional allocation was used to decide how many participants will be interviewed at each facility based on how many patients were seen at each facility. For example the total number of patients seen at the three clinics for a period of time equivalent to the length of study was 141, and number of patients seen at clinic A during the same period was 36. Therefore, the required sample size from clinic A was 36/141*280 = 72 participants.

### Data collection

Selected clinics were visited concurrently, and the number of participants interviewed on each visit depended on the number of mothers seen monthly in each clinic. All mothers in the line, who presented to the clinic each day, meeting the inclusion criteria, were approached by the first author or research assistant and recruited into the study.

Data were collected between December 2021 and February 2022. Data collection was carried out by the author with the help of two nursing assistants as research assistants. The research assistants underwent a 1-day orientation and training by the first author. All mothers in the line coming for review each day were addressed and informed about the purpose of the study by the researcher or trained research assistants. Those consenting to participate in the study were taken to a separate room for an interview after signing an informed consent form. The study adhered to COVID-19 protocols.

On a weekly basis, 10% of completed questionnaires were randomly selected to audit for completeness, missing variables and consistency on days data were collected. Each week after data collection, a team meeting was held virtually and filled questionnaires were cross-checked. Possible non-responses were identified, discussed and noticed.

Translation of all questionnaires was performed by an independent bilingual (Setswana and English) translator and verified by back translation by a different translator who was blinded to the original version. After the back translation, the two versions were compared for any significant differences and the final version was reached through a consensus.

### Measures

A *socio-demographic questionnaire* was used to determine demographic and social factors that possibly predispose mothers to developing PPD. Close-ended questions to determine age, gender, marital status, unplanned pregnancy, number of children, level of education, employment status, history of mental disorder, subjective history of antepartum depression, relationship with partner’s mother, current or history of intimate partner violence and partner support were posed to the participants.

*Edinburg Postnatal Depression Scale* (*EDPS*) is a 10-item, widely used screening scale for PPD in outpatient or home visit settings. It is used to ascertain whether one has depression or not. The scores on each of the 10 items are added up giving a minimum score of 0 and a maximum score of 30.^24^ A cut-off of 10 was used to indicate patients at risk of having PPD. The EPDS has been used and validated in South Africa^25^ and Zimbabwe,^26^ which are similar to our setting.

*Oslo Social Support Scale-3* (*OSSS-3*) was used to assess the level of social support. The OSSS-3 consists of only three items that ask for the number of close confidants, the sense of concern from other people and the relationship with neighbours with a focus on the accessibility of practical help.^27^ The total score ranges from 3 to 14, and the ranges are categorised into poor (3–8), moderate (9–11) and strong (12–14) social support. This instrument has been used previously in Botswana.^28^

### Data analysis

Microsoft Excel was used to capture data and IBM SPSS 28 statistical package version was used to statistically analyse data for the study. The socio-demographic data were analysed by descriptive analysis and expressed as mean, median and standard deviation. Frequency distributions of categorical variables and ranges of continuous variables were calculated. Normally distributed continuous data were summarised using mean and standard deviation. Continuous data that were not normally distributed were summarised using median and interquartile ranges (IQR). Associations between PPD and categorical variables were assessed using chi–square test, Fisher’s exact test or likelihood ratio where applicable. The chi-square test or Fisher’s exact test was used to compare categorical variables and check the bivariate relationship between PPD and relevant risk factors. Risk factors identified in the bivariate analysis were analysed in the multivariable model.

Logistic regression using a forward likelihood ratio approach was conducted to identify associations between PPD and key confounding predictor variables and results were presented in the form of an adjusted odds ratio (AOR). ‘Statistical associations and significance between variables were determined by using a 95% confidence interval, with a significance level of (*p ≤* 0.05)’.^10^

### Ethical considerations

The Botswana Ministry of Health and the University of Botswana Institutional Review Board approved the study on 24 September 2021 and 21 September 2021, respectively (Reference numbers HPDME: 13/18/1 and UBR/RES/IRB/BIO/GRAD/155, respectively). Respondents gave written consent before starting interviews.

## Results

### Participants’ characteristics

A total of 295 postpartum women participated in this study. The median age of participants was 28 years (IQR: 2–33). Most participants (*n* = 154, 52.2%) had planned their pregnancy and more than a third of the mothers were primigravida (*n* = 105, 35.6%).

Antepartum depression was reported by 10.8% (*n* = 32) of participants, while only 1.4% (*n* = 4) participants had a history of being diagnosed with a mental disorder. Based on the OSSS-3, 31.5% (*n* = 93) of participants reported poor social support. Participants’ socio-demographic and clinical characteristics are depicted in Table 1.

**TABLE 1:** Participants’ characteristics, psychosocial factors and social support.

Variable	Category	Frequency (*n*)	%
Maternal age (years)	18–25	106	35.9
	26–43	188	63.7
Marital status	Single	50	16.9
	In a relationship	204	69.2
	Married	41	13.9
Employment status	Employed	109	36.9
	Unemployed	182	61.7
Pregnancy	Planned	154	52.2
	Unplanned	140	47.5
HIV status	Negative	228	77.3
	Positive	57	19.3
Age of the current child (weeks)	6	116	39.3
	8	72	24.4
	12	107	36.3
Level of education	No school attended	4	1.4
	Primary school level	8	2.7
	Secondary school level	195	66.1
	Tertiary level	86	29.2
History of any mental disorder	Yes	4	1.4
	No	291	98.6
History of antepartum depression	Yes	32	10.8
	No	263	89.2
Relationship with partners mother	Good	195	66.3
	Bad	35	11.9
	Not applicable†	64	21.8
Involved in intimate partner violence	Yes	55	18.6
	No	240	81.4
Partner support	Yes	236	80.0
	No	53	18.0
Social support	Poor	93	31.5
	Moderate	152	51.5
	Good	50	16.9

Note: Maternal median age (years): 28; IQR: 23–33. Children median age (years): 2; IQR: 2–4.

† , Neutral.

### Prevalence of postpartum depression

The prevalence of PPD was found to be 33.9% (95% CI–28.5% – 39.6%). A total of 7.8% (*n* = 23) of participants had suicide ideation (thoughts of harming themselves), and 139 (47.1%) of mothers reported having been anxious or worried for no good reason.

### Factors associated with postpartum depression

The majority of the mothers with history of antepartum depression (62.5%), bad relationship with partner’s mother (64.7%), history of intimate partner violence (63.6%) and poor social support (52.7%) screened positive for PPD. History of antepartum depression (*p* = 0.001), bad relationship with partner’s mother (*p* ≤ 0.001), current and past experience of IPV (*p* < 0.001) and social support (*p* ≤ 0.001) were significantly associated with PPD (Table 2). Postpartum depression was, however, not significantly associated with other participants’ characteristics such as age, marital status, planned pregnancy, employment status, HIV status or level of education. This is demonstrated in Table 2 and Table 3.

**TABLE 2:** Association between demographic characteristics of participants and postpartum depression.

Variable	Total		Not depressed		Depressed		Chi Sq Fisher’s E L Ratio	*p*-value
	*n*	%	*n*	%	*n*	%		
**Maternal age (years)**	-	-	-	-	-	-	0.00**	0.989
18–25	106	35.9	70	66.0	36	34.0	-	-
26–43	188	63.7	124	66.0	64	34.0	-	-
**Marital status**	-	-	-	-	-	-	3.52**	0.172
Single	50	16.9	30	60.0	20	40.0	-	-
Relationship	204	69.2	133	65.2	71	34.8	-	-
Married	41	13.9	32	78.0	9	22.0	-	-
**Employment status**	-	-	-	-	-	-	1.09**	0.297
Employed	109	36.9	76	69.7	33	30.3	-	-
Unemployed	182	61.7	116	63.7	66	36.3	-	-
Pregnancy	-	-	-	-	-	-	0.91**	0.341
Planned	154	52.2	106	68.8	48	31.2	-	-
Unplanned	140	47.5	89	63.6	51	36.4	-	-
**Number of children**	-	-	-	-	-	-	1.57***	0.455
1–2	213	72.9	141	48.3	72	24.7	-	-
3–4	72	24.7	48	16.4	24	8.2	-	-
5–7	7	2.4	3	1.0	4	1.4	-	-
**HIV status**	-	-	-	-	-	-	0.66**	0.416
Negative	228	77.3	153	67.1	75	32.9	-	-
Positive	57(19.3)	-	35	61.4	22	38.6	-	-
**Age of the current child (weeks)**	-	-	-	-	-	-	1.02**	0.600
6	116	39.3	81	69.8	35	30.2	-	-
8	72	24.4	46	63.9	26	36.1	-	-
12	107	36.3	68	63.6	39	35.4	-	-
**Level of education**	-	-	-	-	-	-	2.94***	0.401
No school	4	1.4	2	50.0	2	50.0	-	-
Primary school	8	2.7	6	75.0	2	25.0	-	-
Secondary school	195	66.1	123	63.1	72	36.9	-	-
Tertiary level	86	29.2	62	72.1	24	27.9	-	-

Chi Sq, Chi-square test; Fisher’s E, Fisher’s exact test; L Ratio, Likelihood ratio.

** , Chi-square test;

*** , Likelihood ratio.

**TABLE 3:** Association between psychosocial characteristics of participants and postpartum depression.

Variable	Total		Not depressed		Depressed		Chi Sq Fisher’s E L Ratio	*p*-value
	*n*	%	*n*	%	*n*	%		
**History of any mental disorder**	-	-	-	-	-		*	0.505
Yes	4	1.4	2	50.0	2	50.0	-	-
No	291	291.0	193	193.0	98	33.7	-	-
**History of antepartum depression**	-	-	-	-	-	-	13.10**	< 0.001
Yes	32	10.8	12	37.5	20	62.5	-	-
No	263	89.2	183	69.6	80	30.4	-	-
**Relationship with partner’s mother**	-	-	-	-	-	-	17.79**	< 0.001
Good	195	66.3	137	46.6	58	19.7	-	-
Bad	35	11.9	12	4.1	23	7.8	-	-
N/A	64	21.8	45	15.3	19	6.5	-	-
**Intimate partner violence**	-	-	-	-	-	-	26.68**	0.001
Yes	55	18.6	20	36.4	35	63.6	-	-
No	240	81.4	175	72.9	65	27.1	-	-
**Partner support**	-	-	-	-	-	-	9.19**	0.020
Yes	236	80.0	167	70.8	69	29.2	-	-
No	53	18.0	26	49.1	27	50.9	-	-
**Social support**	-	-	-	-	-	-	26.60**	< 0.001
Poor	93	31.5	44	47.3	49	52.7	-	-
Moderate	152	51.5	107	70.4	45	29.6	-	-
Good	50	50.0	44	44.0	6	12.0	-	-

N/A, not applicable (neutral); Chi Sq, Chi-square test; Fisher’s E, Fisher’s exact test; L Ratio, Likelihood ratio.

* , Fisher’s exact test;

** , Chi-square test;

*** , Likelihood ratio.

### Multivariate analysis of factors associated with postpartum depression

Participants with moderate social support (AOR: 5.897, CI: 2.140–16.248, *p* ≤ 0.001) and poor social support (AOR: 2.685, CI: 1.013–7.111, *p* = 0.047) were more likely to be depressed compared to those with good social support. Those with history of intimate partner violence were four times more likely to be depressed (AOR: 4.789, CI: 2.276–10.077; *p* ≤ 0.001) (Table 4).

**TABLE 4:** Multivariate logistic regression of factors associated with postpartum depression.

Variable	Category	AOR	95% CI	*p*-value
**Relationship with partner’s or husband’s mother**				
Good 1 (R)	Bad	2.66	1.08–6.54	0.033
	NA (Undefined)	0.51	0.24–1.09	0.085
**Intimate partner violence**				
Good 0 (R)	No	-	-	-
	Yes	4.79	2.28–10.08	< 0.001
**Social support**				
Good 2 (R)	Moderate	5.90	2.14–16.25	< 0.001
	Poor	2.68	1.01–7.11	0.047

AOR, adjusted odd ratio; R, reference; CI, Confidence interval; NA, not applicable.

Compared to a good relationship with the partner’s or husband’s mother, participants with a bad relationship with the husband’s mother were about two times more likely to develop PPD (AOR: 2.657, CI: 1.080–6.538, *p* = 0.033) (Table 4). Other participants’ characteristics and psychosocial factors did not contribute significantly to the final model fit, hence were not included in the model as predictors of PPD.

## Discussion

This study was carried out to determine the prevalence and risk factors of PPD in women attending under-five wellness clinics in three government clinics in Gaborone. This study showed that the prevalence of PPD is 33.9% in our setting. The study also established that participants who have a history of intimate partner violence, a poor relationship with the partner’s mother or mother-in-law, and a moderate and poor social support were at risk of developing PPD.

The prevalence from our study was similar to the prevalence (33.82%) that was reported in Southwest Ethiopia^7^; however, it is higher than what has been reported globally, in low- and middle-income countries and in other African settings.^5,14,28^ A systematic review and meta-analysis conducted by Hahn-Holbrook J (2018)^5^ revealed a pooled PPD global prevalence of 17.7%, while the pooled prevalence in low-income countries was 25.8% and in middle-income countries was 20.8%. A pooled prevalence of 16.84% was found in Africa as a continent.

African countries such as Ghana^8^ and Sudan^8^ reported rates of PPD occurrence at 7% and 9.2%, respectively, which is lower than the prevalence in our study. The lower prevalence in Sudan could be because of the higher cut-off point of EPDS of 12 compared to 10 used in our study, and participants in the Sudanese study were captured from a tertiary hospital setting, possibly not capturing the population presenting to local clinics. In Ghana, differences in the screening tools and using a population from a hospital setting might explain the difference in the prevalence.

The prevalence in our study was, however, lower when compared to Eswatini 47.4%^12^ and the Republic of South Africa 50.3%.^11^ The differences in prevalence might be because of differences in the types of communities where studies were performed. The majority of reported studies were conducted in rural communities contrary to our study, which was carried out in an urban community.^11,17^ Furthermore, the difference in participants’ characteristics between these studies and our study could explain the difference in prevalence. In our study, participants were of an older age group, with 63.7% being of participants having an age ranging between 24 and 43 years old, while in other studies, most of the participants in studies were below 24 years of age.^11,17^ Pregnancy at a younger age has been found to increase the risk of developing PPD,^10,11,12^ which may explain why prevalence in our setting might be low compared to other studies in Southern Africa. Most participants in our study attained secondary and tertiary education levels (95.2%). Having a low primary level of education has been independently associated with positive screening for PPD^29^ in South Africa, hence lower prevalence in our study when compared to RSA.

History of and current experience of intimate partner violence were found to be a predictor of PPD in this study, with verbal abuse being the most self-reported form of IPV in participants with PPD in this study. These findings are similar to findings in previous studies.^15,16,28,30^ The IPV appears to be a common and important adverse life event placing mothers in the postpartum period at risk of developing PPD. Moreover, pregnancy and postpartum period are vulnerable times placing women at risk of experiencing IPV. Exposure to different forms of IPV such as physical, sexual and economical is more likely to increase the risk of mothers having PPD, and this can be before or after delivery.^28,31^ Therefore, screening for IPV during the antepartum and postpartum period to identify women at risk and provide early intervention can be carried out and hence prevent PPD.

The effects of interpersonal relationship with partner’s mother or mother-in-law play a key role in the mental health of women in the postpartum period. Previous studies have shown that maternal stress because of poor relationships can predispose mothers to PPD.^32^ A good relationship with partners’ mothers directly reduces the risk of PPD because a good relationship is perceived as social support and social support has been shown to be a protective factor against PPD.^33^ Therefore, improving the relationship between new mothers and their mothers-in-law using interpersonal psychotherapy can be considered as an intervention to reduce PPD.^32^

Participants with poor and moderate social support were significantly predisposed to PPD in our study. This finding is similar to findings in other different studies.^17,29,32,35^ Women with good social support have been found to have less severe symptoms of PPD.^37^ Frequent availability of maternal support is associated with a greater sense of community belonging and hence acts as a protective factor and a buffer to developing PPD.^36^ The availability of maternal support is associated with positive mental health among women in the postpartum period. The postpartum period is a time when mothers need support from their family, partner and in-laws, and social support can be used as part of treatment and intervention for PPD. In Botswana, there are cultural practices in our society where relatives come to help mothers after delivery (botsetsi), and this can be encouraged to serve as a form of support to the mother. Primary healthcare workers can counsel new mothers by encouraging traditional postpartum cultural practices as these can possibly decrease PPD in our setting.

We did not find any significant association between unplanned pregnancy and the occurrence of PPD. This was consistent with a study conducted in Eswatini,^12^ but this contrasted with multiple previous studies^7,11,12,29,32^ that showed that unplanned pregnancy is associated with an increased likelihood of developing PPD. The contrast might be explained by the availability of free medical care in Botswana. Mothers with unplanned pregnancies despite their socio-economic status can attend antenatal clinics, receiving management for some complications of pregnancy, health promotion and preventative care necessary to get the mother ready for the newborn child.

History of being diagnosed with a psychiatric disorder did not predict PPD despite being significantly associated with PPD in the bivariate analysis, unlike in other studies.^11,28^ This might have not been evident in the study because of the sampling method used, limiting the ability to detect the statistical significance of these factors. This is because convenient sampling can fail to capture important cases as in under coverage bias typical of this sampling method. The lack of significant association found in this study is however comparative to findings from other studies^7,31^ in similar settings.

Furthermore, there was no significant association between maternal age, marital status, employment status, HIV status, age of current child, number of children and education level. Similar findings were reported in different studies for maternal age and marital status,^15,16^ employment status,^10^ HIV status,^16^ number of children,^15^ age of the current child^15^ and level of education.^16^ This similarity could be because of the similar demographics of participants, that is, women in their childbearing age groups and in the postpartum period.

### Strengths and limitations

This is the first study to determine the prevalence of depression in the postpartum period, and findings from this research can serve as baseline information on PPD for further research to be performed on this topic in our setting. The power of the study was improved by increasing the sample size by adding more participants than the calculated sample size using the Cochrane formula. Two clinics included in the study were in densely populated slums in Gaborone. These areas have many people who have migrated from rural areas to urban Gaborone for employment purposes and have lower rental fees compared to other areas in Gaborone, possibly improving the variability of social status within the participant group in the study.

The findings of this study should be interpreted in the light of some limitations. There was the risk of recall bias when answering questions from EPDS as it required participants to recall events over the past 7 days. Pseudodementia known to occur in patients with depression could have also affected recalling past events when responding to the EPDS questionnaire. Depressed women tend to self-isolate and stay home and may have poor health-seeking behaviour, hence not present to local clinics affecting the overall prevalence. Data collected do not capture mothers who sought medical care from private health facilities. The convenient sampling method used does not favour generalisation of results because participants recruited were readily available and may not be representative of the population being studied. There is an inherent tendency of overestimation in cross-sectional study types, and this is a major limitation.

## Conclusion

There is a high prevalence of PPD in women attending under-five clinics in Gaborone. Moderate and poor social support, experiencing intimate partner violence and bad relationship with partner’s/husband’s mother are significant predictors of PPD development.

Routine screening for IPV and depression symptoms at every opportune antenatal, postnatal and outpatient presentation would enable prevention and early management of PPD. Encouraging an interpersonal relationship psychotherapy focusing on partners’ mothers /mother-in-laws and the new mothers as an intervention for PPD should be recommended. Continued practice of traditional postpartum cultural practices can be recommended to promote social support.

Further research on the prevalence of PPD across the country taking into consideration both rural and urban areas is recommended. Research assessing other types of relationships with the potential to decrease the chances of developing PPD is recommended. Carrying out further research using a prospective study design is recommended.

## References

[1] O’Hara MW, Mc Cabe JE. Postpartum depression: Current status and future directions. Annu Rev Clin Psychol. 2013;9:379–407. 10.1146/annurev-clinpsy-050212-185612 23394227

[2] American Psychiatric Association. Diagnostic and statistical manual of mental disorders. 5th ed. Arlington, VA: American Psychiatric Association; 2013.

[3] Buttner M, O’Hara MW, Watson D. The structure of women’s mood in the early postpartum. Assessment. 2012;19(2):247–256. 10.1177/1073191111429388 22156719

[4] Stewart DE, Vigod SN. Annual review of medicine postpartum depression: Pathophysiology, treatment, and emerging therapeutics. Annu Rev Med. 2019;70:183–196. 10.1146/annurev-med-041217-011106 30691372

[5] Hahn-Holbrook J, Cornwell-Hinrichs T, Anaya I. Economic and health predictors of national postpartum depression prevalence: A systematic review, meta-analysis, and meta-regression of 291 studies from 56 countries. Front Psychiatry. 2018;8:00248. 10.3389/fpsyt.2017.00248 PMC5799244 29449816

[6] O’Hara MW, Swain AM. Rates and risk of postpartum depression – A meta-analysis. Int Rev Psychiatry. 1996;8(1):37–54. 10.3109/09540269609037816

[7] Kerie S, Menberu M, Niguse W. Prevalence and associated factors of postpartum depression in Southwest, Ethiopia, 2017: A cross-sectional study. BMC Res Notes. 2018;11(1):623. 10.1186/s13104-018-3730-x 30157909 PMC6114481

[8] Khalifa DS, Glavin K, Bjertness E, Lien L. Postnatal depression among Sudanese women: Prevalence and validation of the Edinburgh Postnatal Depression Scale at 3 months postpartum. Int J Womens Health. 2015;7:677. 10.2147/IJWH.S81401 26185471 PMC4501244

[9] Anokye R, Acheampong E, Budu-Ainooson A, Obeng EI, Akwasi AG. Prevalence of postpartum depression and interventions utilized for its management. Ann Gen Psychiatry. 2018;17(1):1–8. 10.1186/s12991-018-0188-0 PMC5941764 29760762

[10] Verkuijl NE, Richter L, Norris SA, Stein A, Avan B, Ramchandani PG. Postnatal depressive symptoms and child psychological development at 10 years: A prospective study of longitudinal data from the South African Birth to Twenty cohort. Lancet Psychiatry. 2014;1(6):454–460. 10.1016/S2215-0366(14)70361-X 26361200

[11] Stellenberg EL, Abrahams JM. Prevalence of and factors influencing postnatal depression in a rural community in South Africa. Afr J Prim Health Care Fam Med. 2015;7(1):a874. 10.4102/phcfm.v7i1.874 PMC4729123 26842515

[12] Dlamini LP, Mahanya S, Dlamini SD, Shongwe MC, Shongwe M. Prevalence and factors associated with postpartum depression at a primary healthcare facility in Eswatini. S Afr J Psychiatry. 2019;25:a1404. 10.4102/sajpsychiatry.v25i0.1404 PMC6851868 31745444

[13] Mehta ND, Chen KK, Monzon C, Rosene-Montella K. Common medical problems in pregnancy, postpartum blues, depression, and psychosis. In: McKean SC, Ross JJ, Dressler DD, Scheurer DB, editors. Principles and practice of hospital medicine, 2e. New York, NY: The McGraw-Hill Companies, 2017; p. 1860.

[14] Guintivano J, Manuck T, Meltzer-Brody S. Predictors of postpartum depression: A comprehensive review of the last decade of evidence. Clin Obstet Gynecol. 2018;61(3):591–603. 10.1097/GRF.0000000000000368 29596076 PMC6059965

[15] Dadi AF, Akalu TY, Baraki AG, Wolde HF. Epidemiology of postnatal depression and its associated factors in Africa: A systematic review and meta-analysis. PLoS One. 2020;15(4):e0231940. 10.1371/journal.pone.0231940 32343736 PMC7188237

[16] Barthel D, Kriston L, Fordjour D, et al. Trajectories of maternal ante- and postpartum depressive symptoms and their association with child- and mother-related characteristics in a West African birth cohort study. PLoS One. 2017;12(11):e0187267. 10.1371/journal.pone.0187267 29107975 PMC5673167

[17] January J, Chimbari MJ. Prevalence and factors associated with postnatal depression among women in two rural districts of Manicaland, Zimbabwe. S Afr J Psychiatry. 2018;24:a1176. 10.4102/sajpsychiatry.v24i0.1176 PMC6244063 30473880

[18] Garman EC, Schneider M, Lund C. Perinatal depressive symptoms among low-income South African women at risk of depression: Trajectories and predictors. BMC Pregnancy Childb. 2019;19(1):202. 10.1186/s12884-019-2355-y PMC6570971 31200665

[19] Slomian J, Honvo G, Emonts P, Reginster JY, Bruyère O. Consequences of maternal postpartum depression: A systematic review of maternal and infant outcomes. Women’s Health. 2019;15:1745506519844044. 10.1177/1745506519844044 PMC6492376 31035856

[20] Grace SL, Evindar A, Stewart DE. The effect of postpartum depression on child cognitive development and behavior: A review and critical analysis of the literature. Arch Womens Ment Health. 2003; 6:263–274. 10.1007/s00737-003-0024-6 14628179

[21] Statistics Botswana. 2022 Population and housing census preliminary results V2 [serial on the Internet]. 2022 [cited 2022 May 10]. Available from: https://www.statsbots.org.bw/sites/default/files/2022%20Population%20and%20Housing%20Census%20Preliminary%20Results.pdf

[22] Cochran WG. Sampling techniques. 3rd ed. New York, NY: John Wiley & Sons, 1977; p. 428.

[23] Prevention of mother to child Transmission program. Annual greater Gaborone District Health Management Team PMTCT statistics. Gaborone: Ministry of Health; 2021.

[24] Cox JL, Holden JM, Sagovsky R. Detection of postnatal depression: Development of the 10-item Edinburgh Postnatal Depression Scale. Br J Psychiatry. 1987;150(6):782–786. 10.1192/bjp.150.6.782 3651732

[25] Lawrie T, Hofmeyr G, De Jager M, Berk M. Validation of the Edinburgh Postnatal Depression Scale on a cohort of South African women. S Afr Med J. 1998;88:1340–1344. 9807193

[26] Chibanda D, Mangezi W, Tshimanga M, et al. Validation of the Edinburgh Postnatal Depression Scale among women in a high HIV prevalence area in urban Zimbabwe. Arch Womens Ment Health. 2010;13(3):201–206. 10.1007/s00737-009-0073-6 19760051

[27] Kocalevent RD, Berg L, Beutel ME, et al. Social support in the general population: Standardization of the Oslo social support scale (OSSS-3). BMC Psychol. 2018;6(1):31. 10.1186/s40359-018-0249-9 30016997 PMC6050647

[28] Olashore AA, Molebatsi K, Musindo O, et al. Psychosocial predictors of anxiety and depression in a sample of healthcare workers in Botswana during the COVID-19 pandemic: A multicenter cross-sectional study. SAGE Open Med. 2022;10:205031212210850. 10.1177/20503121221085095 PMC8941707 35342632

[29] Dadi AF, Miller ER, Mwanri L. Postnatal depression and its association with adverse infant health outcomes in low- and middle-income countries: A systematic review and meta-analysis. BMC Pregnancy Chidb. 2020;20:416. 10.1186/s12884-020-03092-7 PMC7374875 32698779

[30] Phukuta NSJ, Omole OB. Prevalence and risk factors associated with postnatal depression in a South African primary care facility. Afr J Prim Health Care Fam Med. 2020;12(1):a2538. 10.4102/phcfm.v12i1.2538 PMC7736693 33354984

[31] Howard LM, Molyneaux E, Dennis CL, Rochat T, Stein A, Milgrom J. Non-psychotic mental disorders in the perinatal period. Lancet. 2014;384(9956):1775–1788. 10.1016/S0140-6736(14)61276-9 25455248

[32] Abdollahi F, Zarghami M, Azhar MZ, Sazlina SG, Lye MS. Predictors and incidence of postpartum depression: A longitudinal cohort study. J Obstet Gynaecol Res. 2014;40(12):2191–2200. 10.1111/jog.12471 25132641

[33] DeMontigny F, Gervais C, Pierce T, Lavigne G. Perceived paternal involvement, relationship satisfaction, mothers’ mental health and parenting stress: A multi-sample path analysis. Front Psychiatry. 2020;11:578682. 10.3389/fpsyt.2020.578682 33240130 PMC7667046

[34] Qi W, Liu Y, Lv H, et al. Effects of family relationship and social support on the mental health of Chinese postpartum women. BMC Pregnancy Childb. 2022;22(1):1–10. 10.1186/s12884-022-04392-w PMC8787939 35078423

[35] Hain S, Oddo-Sommerfeld S, Bahlmann F, Louwen F, Schermelleh-Engel K. Risk and protective factors for antepartum and postpartum depression: A prospective study. J Psychosom Obstet Gynecol. 2016;37(4):119–129. 10.1080/0167482X.2016.1197904 27376660

[36] Varin M, Palladino E, Orpana HM, et al. Prevalence of positive mental health and associated factors among postpartum women in Canada: Findings from a national cross-sectional survey. Matern Child Health J. 2020;24(6):759. 10.1007/s10995-020-02920-8 32323116 PMC7198477

[37] Pao C, Guintivano J, Santos H, Meltzer-Brody S. Postpartum depression and social support in a racially and ethnically diverse population of women. Arch Womens Ment Health. 2019;22(1):105. 10.1007/s00737-018-0882-6 29968129 PMC6800248

